# Adapting the McMaster-Ottawa scale and developing behavioral anchors for assessing performance in an interprofessional Team Observed Structured Clinical Encounter

**DOI:** 10.3402/meo.v20.26691

**Published:** 2015-05-22

**Authors:** Désirée Lie, Win May, Regina Richter-Lagha, Christopher Forest, Yvonne Banzali, Kevin Lohenry

**Affiliations:** Department of Family Medicine, Keck School of Medicine of the University of Southern California, Los Angeles, CA, USA

**Keywords:** interprofessional education, observed structured clinical encounter, assessment, standard-setting

## Abstract

**Background:**

Current scales for interprofessional team performance do not provide adequate behavioral anchors for performance evaluation. The Team Observed Structured Clinical Encounter (TOSCE) provides an opportunity to adapt and develop an existing scale for this purpose. We aimed to test the feasibility of using a retooled scale to rate performance in a standardized patient encounter and to assess faculty ability to accurately rate both individual students and teams.

**Methods:**

The 9-point McMaster-Ottawa Scale developed for a TOSCE was converted to a 3-point scale with behavioral anchors. Students from four professions were trained *a priori* to perform in teams of four at three different levels as individuals and teams. Blinded faculty raters were trained to use the scale to evaluate individual and team performances. G-theory was used to analyze ability of faculty to accurately rate individual students and teams using the retooled scale.

**Results:**

Sixteen faculty, in groups of four, rated four student teams, each participating in the same TOSCE station. Faculty expressed comfort rating up to four students in a team within a 35-min timeframe. Accuracy of faculty raters varied (38–81% individuals, 50–100% teams), with errors in the direction of over-rating individual, but not team performance. There was no consistent pattern of error for raters.

**Conclusion:**

The TOSCE can be administered as an evaluation method for interprofessional teams. However, faculty demonstrate a ‘leniency error’ in rating students, even with prior training using behavioral anchors. To improve consistency, we recommend two trained faculty raters per station.

Interprofessional education (IPE), defined most commonly as ‘occasions when two or more professions learn with, from and about each other to improve collaboration and the quality of care’ (1) has received increasing attention in health sciences education. Models of IPE delivery within undergraduate and graduate education involving up to six professions have been reported (2, 3). These models include the use of patient simulations for teaching (4, 5). Guidelines for curricula to teach desired IPE competencies have proliferated in recent years (6–9). However, various reviews (10–13) consistently emphasize the need for theoretical frameworks to underpin IPE outcomes research design, to address the inherent complexity of IPE and the influence of learners, curriculum format and timing, faculty abilities and organizational context on learning (14). IPE outcomes research has focused on changes in learner attitudes, knowledge, and collaborative behaviors, mostly in the short term (11). There remains a need for standard-setting and tools that accurately measure and reflect student performance in teams that have potential to be applied to clinical practice settings (15).

Assessment tools that are currently used include attitude measures such as the Readiness for Interprofessional Learning Scale (16) or the Interdisciplinary Education perception Scale (17), and tools such as the TeamSTEPPS communication behaviors and assessment instruments (18, 19), all relying on self-assessment. Validated tools that allow independent observer ratings based on objective assessment and documentation of individual and team behaviors are needed to add rigor to the evaluation process.

The Objective Structured Clinical Examination (OSCE) has been used in health professions education as a valid and reliable method for assessing student knowledge and skills through structured observation and the use of standardized patients (SPs) and observer checklists (20, 21). Step 2 of the United States Medical Licensing Examination Clinical Skills Examination has used SPs to ‘test medical graduates on their ability to gather information from patients, perform physical examinations, and communicate their findings to patients and colleagues’ (www.usmle.org/step-2-cs/). For interprofessional learning, the 9-point McMaster-Ottawa scale (22) was developed as a checklist with the purpose of allowing observing raters to assess team and individual performance using six core IPE constructs. These constructs are communication, collaboration, roles and responsibilities, collaborative patient-centered approach, conflict management, and team functioning (23, 24). The face and content validity of the Team Observed Structured Clinical Encounter (TOSCE) was established and 10 TOSCE topics were selected for development (25, 26). The TOSCE purports to evaluate individual and team performance in settings ranging from maternity (27, 28) to palliative care (29). However, we were unable to find specific behavioral anchors for rating individual and team behaviors; this creates a challenge for educators attempting to apply the scale in either a standardized simulated or a real clinical setting.

We, therefore, conducted a study to develop standardized behavioral anchors for faculty to rate individual students and interprofessional team performance, using the six McMaster-Ottawa constructs; and to train faculty to use the scale. Our two aims were first, to assess the feasibility of using the retooled scale in a TOSCE setting; and second, to evaluate the ability of faculty raters to use the retooled scale to accurately distinguish different levels of student and team performance. We hypothesized that faculty raters would be able to accurately rate up to four students within an IPE team as well as overall team performance in a 35-min encounter. We also hypothesized that faculty would be able to identify high and low-performing individuals and teams but would have greater difficulty accurately discriminating levels of individual performance in teams with mixed individual performance levels.

The Institutional Review Board of the University of Southern California approved the study.

## Methods

### Study setting

Our study was conducted at the health science campus of a single institution (the University of Southern California) located in urban Los Angeles, California.

### Study participants

Participants were 16 volunteer faculty members representing dentistry, medicine, occupational therapy, pharmacy, and physician assistant professions with experience teaching and assessing students, and no prior experience with IPE assessment. Faculty members were trained as raters immediately prior to the TOSCE administration and were blinded to the purpose of the study, as well as student and IPE team performance levels. We trained volunteer students from a student-run IPE clinic in teams to perform at different levels to assess how well the scale allowed trained, blinded faculty raters to discriminate among the different performance levels. Four SPs were recruited from a database of experienced SP actors to perform at TOSCE stations.

### Development of behavioral anchors

Three authors (DL, WM, and RR) examined the six constructs from the McMaster-Ottawa scale and the descriptors associated with each. They determined *a priori* that it was not feasible to develop anchors for the original 9-point scale as it was extremely difficult to distinguish and describe nine different levels of behaviors for individual students. Through an iterative process of discussion, consensus-building and review, three levels of performance were judged as capable of being distinguished. Level 3 was defined as the highest, or ‘above expected’; 2 as the intermediate, or ‘at expected’; and 1 as the lowest, or ‘below expected’ level. A detailed description of observable teamwork behaviors for each level of individual performance was created, with a final total of 18 (6×3) non-overlapping behavior categories (Table 1).

**Table 1:** Modified McMaster-Ottawa scale for rating individual students, with instructions for 3-point scoring, Keck School of Medicine of the University of Southern California, 2014

	Individual rating		
	
Competencies	Below Expected	At Expected	Above Expected
Communication Assertive communication Respectful communication Effective communication	1	2	3
Collaboration Establishes collaborative relationships Integration of perspectives Ensures shared information	1	2	3
Roles and responsibilities Describes roles and responsibilities Shares knowledge with others; accepts accountability	1	2	3
Collaborative patient–family centered approach Seeks input from patient and family Shares with patients and family Advocates for patient and family	1	2	3
Conflict management/resolution Demonstrates active listening Respectful of different perspectives Works with others to prevent conflict	1	2	3
Team functioning Evaluates team function and dynamics Contributes effectively Demonstrates shared leadership	1	2	3
Global rating score	1	2	3

Instruction to rater: Observe students during the huddles and patient encounter. Using the 3-point scale, assess *each* student's demonstration of the six competencies; provide an overall global score based on all the competencies. Please score all behaviors. Do not leave any item blank. **Detailed explanation of performance behaviors for each category:** **Communication:** ***Above expected:*** (The student) expresses opinions in an objective, confident manner; speaks calmly in disagreements; shows deference; listens carefully; asks clarifying questions; responsive to non-verbal clues. ***At expected:*** speaks politely; able to comfortably express disagreement and share opinions; does not talk down to others; fully attentive to others’ non-verbal clues. ***Below expected:*** expresses opinions in a hostile manner; talks down to others; does not make good eye contact or adopt a listening posture. **Collaboration:** ***Above expected:*** (The student) incorporates information provided by others; ensures information is disseminated to the entire team. ***At expected:*** uses information provided by team members. ***Below expected:*** does not use information provided by members. **Roles/responsibilities:** ***Above expected:*** (The student) shows initiative in describing own role/scope; explicitly asks for and clarifies members’ roles/responsibilities; describes contributions of other professions’ to the team's task; uses evidence-based practice to inform actions; clearly describes the rationale and takes responsibility for own challenging/blameworthy actions. ***At expected:*** articulates own role and work when asked; inquires about team members’ roles/responsibilities; shares evidence-based practice; describes actions. ***Below expected:*** does not ask for roles/responsibilities of others; does not take ownership of decisions; if challenged, is vague in description of actions. **Collaborative patient-family centered approach:** ***Above expected:*** (The student) provides patient/family with options for care and reviews including pros/cons; actively summarizes and attempts to incorporate family members’ views in care plans. ***At expected:*** listens/solicits family members’ views; provides patient/family with options for care; articulates these needs to the team. ***Below expected:*** ignores the family's views/needs, fails to provide options for care. **Conflict management resolution:** ***Above expected:*** (The student) seeks harmony by listening respectfully to all; acknowledges and processes conflict; initiates resolution, seeks consensus, respects differing opinions; develops common agreement. ***At expected:*** listens to team members, asks for feedback, recognizes conflict but does not develop common agreement. ***Below expected:*** ignores and interrupts team members, avoids acknowledging conflict. **Team functioning:** ***Above expected:*** (The student) discusses how the team can be more effective; keeps the climate for team functioning constructive; contributes to discussion; encourages others to contribute; takes a leadership role; allows others to lead when appropriate. ***At expected:*** observes team dynamics and determines the climate for the team's functioning; contributes to the discussion. ***Below expected:*** does not determine the team climate; fails to contribute to the discussion; states views but does not engage in dialog. **Global rating score:** Provide a single rating of the student's performance based on all the ratings above.

The same authors developed anchors for the team rating to evaluate team-level performance separate from individual-level performance. We based anchors on factors reported as associated with better patient outcomes (30). Effective team performance was evaluated based on the perception of the level of care afforded the patient due to the team acting as an integrated whole (Table 2).

**Table 2:** Modified McMaster-Ottawa scale for rating teams, with instructions for 3-point scoring, Keck School of Medicine of the University of Southern California, 2014

	Individual rating		
	
Competencies	Below Expected	At Expected	Above Expected
Communication (with patient) Members demonstrate assertive communication Members demonstrate respectful communication Members demonstrate effective communication	1	2	3
Collaboration (within the team) Establishes collaborative relationships Integration of perspectives Ensures shared information	1	2	3
Roles and responsibilities Members describe roles and responsibilities Members share knowledge with each other; accepts to one another accountability	1	2	3
Collaborative patient–family centered approach Members seek input from patient and family Members share information with patients and family Members advocate for patient and family	1	2	3
Conflict management/resolution (within the team) Members demonstrate active listening Members share different perspectives Members work with each other to prevent conflict with one another	1	2	3
Team functioning Members evaluate team function and dynamics Members contribute effectively Members demonstrate shared to team function leadership	1	2	3
Global rating score	1	2	3

Scoring instruction to rater: Observe the team interaction at the pre- and post-encounter huddle and the patient encounter. Do not interrupt the team. Using the 3-point scale, assess the team's performance (regardless of the individuals’ performance) in each of the six competencies and provide an overall/global score based on all these factors. **Detailed explanation of team behaviors for each category:** **Communication:** ***Above expected:*** (The team) provides comprehensive information about the purpose of the encounter and its findings; anticipates the patient's questions by asking for questions; addresses concerns and answers questions directly; is explicit about conversations among the members; and includes the patient in those discussions. ***At expected:*** provides basic information about the purpose of the encounter; respectfully addresses the patient's questions when initiated by the patient; and includes the patient in its discussions. ***Below expected:*** fails to inform the patient of its actions and intentions; talks down to the patient and/or avoids dialog even when questioned by the patient; ignores the patient when conversing with one another. **Collaboration:** ***Above expected:*** (The team) recognizes disagreements and acts to reach consensus so that the patient perceives a unified approach. ***At expected:*** is able to reach agreement by discussing issues in the patient's best interests. ***Below expected:*** is unable to reach agreement on at least half the issues prior to or after the patient encounter. **Roles and responsibilities:** ***Above expected:*** (The team) members actively solicit information about one another's roles before the patient encounter. ***At expected:*** members check in when a misunderstanding about one another's roles occurs. ***Below expected:*** members act on mistaken assumptions about one another's roles. **Collaborative patient-family centered approach:** ***Above expected:*** (The team) elicits family and community information, and actively seeks to involve both in the patient's care plan. ***At expected:*** (The team) elicits some family or community information. ***Below expected:*** (The team) fails to elicit any information about the patient's family or home setting. **Conflict management resolution:** ***Above expected:*** (The team) recognizes areas of potential conflict and elicits ways to resolve them; and agrees on a process to anticipate future conflict. ***At expected:*** members listen to one another, ask for feedback if not clear and recognize conflict. ***Below expected:*** members argue in front of the patient with no mechanism for resolving the arguments. **Team functioning:** ***Above expected:*** (The team) is able to reflect on its own actions and purpose and change dynamics to achieve excellence in team function. ***At expected:*** demonstrates recognition of its function as a unit and discusses communication strategies. ***Below expected:*** has no recognition of the need to function as a unit; individuals make decisions according to their own opinion. **Global rating score:** Provide an overall rating for the team's performance based on all the factors above.

### Study design and TOSCE administration

This is an exploratory and feasibility study for scale development and implementation. One TOSCE station (case available on www.fhs.mcmaster.ca/tosce/en/tosce_stations.html) was selected and modified by consensus agreement among the authors representing the four student professions involved (medicine, physician assistant, pharmacy and occupational therapy). The case selected (stroke) was deemed to be at an appropriate difficulty level to involve all four professions and capable of testing team and individual behaviors. The case was that of a hospitalized rehabilitating patient with hemiplegic stroke who now requests discharge 1 week after admission. Case instructions required the students to *use skills specific to your own discipline and knowledge of others on your healthcare team, to assess the patient's needs and develop a care plan for him*. The team communicated only with the patient who was in a wheelchair and who had spousal support at home. The spouse was not present for the encounter. The timeframe of 35 min for the station was based on the published recommendation (www.fhs.mcmaster.ca/tosce/en/toolkit_guidelines.html).

Our focus was on potential differences among faculty in rating students and teams, so it was imperative that we distinguish variation in student scores attributable to raters from variation attributable to station differences. Due to constraints of available faculty time (4 hours) and the length of each TOSCE station (35 minutes), limiting the study to one station (stroke) allowed us to determine variation due to raters alone. We anticipate future research to examine whether or not station differences affect faculty ratings of students and teams.

One week before the TOSCE was administered, the four student teams (teams A, B, C and D) were trained by three authors (DL, CF, KL) to perform at different skill levels. The students portrayed health professions trainees at the beginning of their clinical training. Team A consisted of four level 3 (above expected) students, team B consisted of two level 3 students and two level 2 (at expected) students, team C consisted of two level 1 (below expected) students and two level 2 students, and team D consisted of three level 1 students and one level 2 student. In each team, the lowest-performing student was chosen to be from a different profession. Team A was trained to portray a team functioning ‘above expected’, team B ‘at expected’, team C ‘at expected’ and team D ‘below expected’. Training of students occurred over 3 h with the use of the retooled behavioral anchors (Table 1) and video demonstrations, followed by practice and feedback from other team members and trainers. Students practiced until the trainers were able to distinguish levels of performance in a mock patient encounter. The faculty trainers did not participate as raters in the actual TOSCE.

Blinded faculty raters were told at recruitment that no prior experience for rating IPE team performance was necessary. They were *not* informed that students had been trained to perform at different levels of performance until after the TOSCE was completed. They received 60 min of training immediately prior to TOSCE administration. Training consisted of independent review of the retooled scale and anchors and group discussion, followed by a viewing of the same video demonstrations representing three different levels of performance that were shown to the trained student teams. Faculty trainers (DL, CF, KL) stressed that the rating scale assessed only performance related to team behaviors, and not the competency of the students within their own particular professions. Training was deemed to be completed when all 16 raters agreed on the performance level of students and teams shown in the videos. There were four faculty raters from different professions at each TOSCE station. Each rater remained at their one assigned station, thus rating all four teams (16 students representing all three levels of individual and team performance) that rotated through their station. The raters were instructed not to communicate with one another. Faculty observed teams without intrusion, and sat 8–12 feet away from the teams and SP. They were given 10 min to complete ratings after 35 min of observation. Of the 35-min encounter, 5 min were spent on the pre-huddle, 20 min with the SP and the final 10 min in a post-encounter debrief. The team pre-huddle and debrief took place in a room adjacent to the patient encounter. The faculty followed and observed the team during the entire 35 min while the SP had access to the team only during the 20 min of his case performance. A post-TOSCE survey was administered to raters regarding the feasibility of the TOSCE and its utility as a teaching and evaluation tool. At the end of the TOSCE, after all rating forms and surveys were collected, raters were debriefed and the ‘correct’ performance level of each team and student revealed. All encounters and team interactions were videotaped.

### Data collection

Rating forms were completed in hard copy. Each rater completed 20 rating forms (16 for individual students and four for the four teams). Post-TOSCE surveys were collected from all raters. De-identified data were entered into Excel format.

### Data analysis

Descriptive statistics were used to examine faculty ability to accurately distinguish students and teams performing below, at, and above expectation to assess the feasibility and utility of using such a scale for formative evaluation. For each faculty rater, we constructed student mean performance scores across the six competencies and compared those values to assigned student levels of performance. Individual and team scores and post-TOSCE survey responses were analyzed using SPSS and GENOVA (31).

A generalizability study (G-study) was conducted to examine variability in student scores due to faculty variation as opposed to other sources of error variation. Generalizability theory (G-theory) allows us to disentangle variation in student performance scores due to different sources of measurement error (32, 33), such as those attributable to item, station, or rater, and the interactions between them. According to G-theory, variation in student TOSCE performance scores can be deconstructed into person (p) variation, or the variation in examinee ability; and error variation, due to various sources of measurement error, known as facets. Of interest to us, then, is the calculation of variation in scores, or variance components, attributable to each of these facets. Our G-study investigated the relative influence of faculty rater (*r*) as well as the interaction of person-by-rater (*pr*). Of particular interest to our study, was the proportion of measurement error in student scores and in faculty accuracy, or ability to correctly identify student performance levels, attributable to trained raters.

## Results

### TOSCE administration and feasibility

All 16 faculty raters received 60 min of pre-TOSCE training until they reported sufficient familiarity with the scale anchors to begin actual rating. Faculty blinding was successful for 13 raters. Three raters suspected some student pre-training after observing two teams, and reported afterwards that they simply continued rating without any effect on their perception of student or team performance. The remaining raters did not suspect during the TOSCE that the students had been pre-trained. The individual students and teams were observed on remote cameras, and were deemed to be performing at their assigned levels by faculty trainers who rated their performance and provided feedback as needed between station performances. All 16 raters were able to complete five ratings per encounter within the allotted time. A total of 320 rating forms were collected. There were no significant logistic issues.

The post-TOSCE survey response rate was 100% (*N=*16). On a scale of 1 (strongly agree) to 5 (strongly disagree) faculty believed (i.e., percentage who agreed or strongly agreed) they had adequate time to rate a maximum of four students per station (94%). Faculty agreed/strongly agreed that the TOSCE was useful for assessing individual (81%) and team (81%) performance. Faculty agreed/strongly agreed that this experience made them more competent to rate team skills (81%) and that the TOSCE should be offered as part of IPE curricula (69%). Despite their training, faculty were ‘not highly confident’ about their rating scores for individuals (50% agreed/strongly agreed); however, they expressed ‘high confidence’ on their scores for teams (75% agreed/strongly agreed). Some expressed a need for more training and a simpler rating form in their comments.

### Faculty rating ability

Though 16 faculty participated, subsequent analysis of the data utilized scores from only 15; data from one was excluded due to failure to follow directions. Four raters neglected to furnish scores on one or two competencies for some students. Results, however, did not change substantially when data from these raters were excluded. Therefore, when constructing average student performance level scores, data from these raters were included. Table 3 displays faculty ability to correctly identify student performance levels.

**Table 3:** Correct and incorrect identification of student performance levels for the TOSCE by faculty rater, Keck School of Medicine of the University of Southern California, 2014

	No. of students							
	
	Students portraying ‘below expected’ (level 1) *N=*5		Students portraying ‘at expected’ (level 2) *N=*5		Students portraying ‘above expected’ (level 3) *N=*6			
					
Faculty	Correct	Incorrect	Correct	Incorrect	Correct	Incorrect	Total correct *n* (%)	Total in-correct *n* (%)
1	1	4	5	0	4	2	10 (63)	6 (38)
2	4	1	4	1	5	1	13 (81)	3 (19)
3	1	4	2	3	5	1	8 (50)	8 (50)
4	3	2	3	2	4	2	10 (63)	6 (38)
5	0	5	5	0	5	1	10 (63)	6 (38)
6	2	3	5	0	2	4	9 (56)	7 (44)
7	4	1	1	4	1	5	6 (38)	10 (63)
8	1	4	3	2	2	4	6 (38)	10 (63)
9	3	2	4	1	5	1	12 (75)	4 (25)
10	2	3	2	3	5	1	9 (56)	7 (44)
11	3	2	2	3	3	3	8 (50)	8 (50)
12	2	3	5	0	5	1	12 (75)	4 (25)
13	2	3	4	1	3	3	9 (56)	7 (44)
14	3	2	4	1	4	2	11 (69)	5 (31)
15	3	2	5	0	5	1	13 (81)	3 (19)
Average^a^	2.3 (46)	2.7 (54)	3.6 (72)	1.4 (28)	3.9 (65)	2.1 (35)	9.7 (60.8)	6.3 (39.2)

a Average indicates the average number (%) of students within each performance level category.

TOSCE: Team Observed Structured Clinical Encounter.

Some faculty were more accurate than others, evidenced by a range (Table 3) in the number of correctly identified performance level of students, from 6 (38%) to 13 (81%). No faculty correctly identified the performance level of all 16 students. The average number of students correctly and incorrectly identified by performance level by faculty revealed that correctly identifying students performing ‘below expected’ was the most difficult for faculty. In fact, more students portraying ‘below expected’ performance on average were scored by faculty as performing ‘at expected’ or even, in some instances, ‘above expected’ (*M*=2.7, or 54% of students) than at their correct performance level (*M*=2.3, or 46% of students). Faculty were on average more accurate in their designation of students performing at (*M=*3.6, or 72% of students) and above (*M=*3.9, or 65% of students) expectation. For team performance, individual faculty accurately rated 50–100% of team performances. Faculty were more accurate in assessing the level of team performance for the high- and low-performing teams (88% correct for the ‘above expected’; 100% correct for the ‘below expected’ teams) and less accurate with ‘at expected’ teams (50% correct; with 50% incorrectly rated as ‘below expected’).

### G-study findings and implications

We performed a G-study to examine the variation in student scores attributable to faculty alone and to the interaction of student and faculty. Table 4 displays estimated variance components of these various sources of measurement error, or facets, in student scores, and provides G-study results for a TOSCE involving one, two and four faculty raters. Because students were assigned specific levels of performance, it is important to note that we cannot draw any conclusions from these calculations about the variation in student ability captured by TOSCE scores. Though our calculations for a four-faculty TOSCE – in which each student is scored by four faculty raters – indicated that the level of student performance differed substantially between students with over 80% of the total variance attributable to systematic differences between students, this variation is ‘manufactured’ because our trained students were assigned in nearly equal numbers to portray all three performance levels. Our calculations for a one-station TOSCE involving four faculty rating students on six competencies indicated a small percentage (nearly 4%) of variation in student scores were attributable to faculty rater (0.01058), indicating that compared to one another, no faculty rater was more lenient or strict than another. A very small percentage (0.15%) of variation was attributable to competency (0.00042), indicating that the six competency categories were equally difficult for students. We attributed a larger proportion (about 11%) of the variance in scores to the interaction between person, or student, and rater (0.03061) suggesting that the relative standing of students may vary from rater to rater. In a TOSCE involving two raters, the percent of total variance attributable to the interaction of student and rater was, as expected, even higher (0.06122) at about 18%.

**Table 4:** Estimated variance components for student performance scores on TOSCE, Keck School of Medicine of the University of Southern California, 2014

Source of variance	df^a^	1 faculty rater 6 competencies^b^	2 faculty raters 6 competencies^b^	4 faculty raters 6 competencies^b^
Student (*p*)	15	0.23441 (55.00)	0.23441 (69.86)	0.23441 (82.75)
Faculty (*r*)	10	0.04234 (9.93)	0.02117 (6.31)	0.01058 (3.73)
Competency (*c*)	5	0.00042 (0.10)	0.00042 (0.13)	0.00042 (0.15)
*pr*	150	0.12243 (28.72)	0.06122 (18.25)	0.03061 (10.81)
*pc*	75	0.00081 (0.19)	0.00081 (0.24)	0.00081 (0.29)
*rc*	50	0.00102 (0.24)	0.00051 (0.15)	0.00025 (0.09)
*prc, e*	750	0.02480 (5.82)	0.01240 (3.70)	0.00620 (2.19)

a df indicates degrees of freedom.

b Variance component (% of total variance).

We also conducted a G-study to examine variation in faculty ability to correctly identify student performance levels using faculty accuracy scores, based on the comparison of faculty average student scores to assigned student performance levels. Faculty were either ‘correct’ or ‘incorrect’ in their assessment of student performance level. Table 5 displays these results. In this analysis, our calculations for a four-team TOSCE, in which students are ‘nested’ within teams, showed variation in faculty ability to accurately score student performance. Nearly 25% of the total variance in faculty accuracy scores was attributable to systematic differences between faculty raters. A moderate percentage of variation in faculty accuracy was attributable to the interaction of faculty and team (0.00487, or about 19%), indicating that the relative accuracy of faculty raters may vary from student team to student team. Additionally, there was a large percentage (nearly 34%) of variation in faculty accuracy attributable to the interaction of faculty rater, student nested within team (*s:t*) commingled with random error (0.00883). These results reaffirmed the need to address the potential impact of faculty–student and faculty–team interactions on performance scores when administering the TOSCE.

**Table 5:** Estimated variance components for faculty ability to correctly identify student performance level on TOSCE, Keck School of Medicine of the University of Southern California, 2014

Source of variance	df^a^	1 team, 1 student/team^b^	4 teams, 4 students/team^b^
Faculty (*p*)	14	0.00650	0.00650 (24.81)
Team (*t*)	3	0.00620	0.00155 (5.92)
Student (*s*): Team (*t*)	12	0.07123	0.00445 (16.98)
*pt*	42	0.01949	0.00487 (18.59)
*ps:t,e*	168	0.14127	0.00883 (33.70)

a df indicates degrees of freedom.

b Variance component (% of total variance).

TOSCE: Team Observed Structured Clinical Encounter.

## Discussion

We conducted a study to examine the feasibility of conducting a TOSCE using a retooled McMaster-Ottawa scale with behavioral anchors to standardize observer ratings. We offered the ideal conditions under which the scale could perform, by providing variability for all three levels of performance among the students and teams, as well as pre-training faculty to rate using the retooled scale. We found that students and teams could be rated by trained faculty within a 35-min encounter. We met our hypothesis that faculty were able to distinguish the lowest and highest levels of performance for both individuals and teams. We found that errors in rating students tended to occur in the direction of over-rating student performance. In other words, faculty tended to assign higher levels of performance even when observing lowest-level performance behaviors, that is, they demonstrated the ‘leniency error’ documented in other evaluation studies (34, 35). To reduce such errors in real-life assessment, we recommend either Rater Error Training or Frame-of-Reference Training with an emphasis on an increase in the number of observations especially for lower-performing students (36). Error Rater Training seeks to improve the accuracy of ratings by correctly identifying and decreasing common ‘rater biases’ or ‘rater errors’ due to factors such as leniency or central tendency. Frame-of-reference training refers to using a reference point to provide a match between the rater's scores and the ratees’ true scores, and relies on the content rather than the process of rating to reduce rater bias.

In addition, other studies (37, 38) found that observers had difficulty distinguishing among 11 team competencies and recommended that researchers use the simplest factor structure when assessing team work. In our TOSCE, there were six team competencies that could have contributed to the challenge of accurate rating. Future studies using more stations and raters should permit factor analysis with the aim of further simplifying the scale structure. Some of our variation in faculty ability to accurately assess individual-level performance may also have been due to inadequate rater training. We found that having more than one rater increased rating reliability. This is similar to the findings of Hull (39) where high inter-observer agreement was reached with two trained raters for the Observational Teamwork Assessment for Surgery with five teamwork behaviors.

In our study, students were assigned in nearly equal numbers to portray all three performance levels, leading to an unusually high level of variation in student ability. Were we to administer the TOSCE to students in the real world, we would very likely not achieve similar results in terms of faculty discrimination. The attributable student-rater variance we found (11% for one rater and 18% for two raters) suggests that to ensure adequate reliability, we would likely need more than one faculty rater in each station were we to administer the TOSCE to untrained (i.e., real world) students.

We purposefully limited our study to assessing faculty rating accuracy by excluding the effect of the clinical station on the retooled scale and to permit more rigorous examination of the scale in the real world setting. Our study has several strengths. One is that quality of student performance was tightly controlled by training and observation of performance during the TOSCE. Another is the use of G-theory to examine relative sources of error in student performance scores. Although three of the blinded raters were able to guess that students had been pre-assigned to perform at different levels, they were not influenced by this suspicion in their ratings. One study limitation is that the proportion of lowest-performing students was one-third in our study, a ratio much higher than usually seen in health professions education. Another limitation is the small number of raters and teams, due to the time constraint of completing the study within a 4-h timeframe. Future research should examine the impact of station differences on rating accuracy, and involve higher numbers of faculty raters, with the inclusion of raters from other professions.

## Conclusion

Use of the adapted TOSCE scale with behavioral anchors is feasible when administered to an interprofessional team of up to four students. Faculty pre-training allows for evaluation of performance. We recommend that a team of at least two faculty raters be assigned per station, to more accurately rate individuals, and that more focused training be provided to address the tendency for faculty to avoid scoring students poorly.
